# Biological Effects of Selenium-Containing Ionic Liquids: Cytotoxicity, Mercury Interaction, and Antiproliferative Activity in Glioblastoma Cells

**DOI:** 10.1002/jat.70280

**Published:** 2026-07-08

**Authors:** Guilherme Schmitt Rieder, Alessandro de Souza Prestes, Fernanda D’Avila da Silva, Nilda de Vargas Barbosa, Alix Y. Bastidas Ángel, Mariana F. Soares, Carolina Martins Fernandes, Maria Anttônia Oliveira Petersen, Manuela Moura Silva, Juliete Nathali Scholl, Fabrício Figueiró, Pablo Andrei Nogara, Laura Orian, Eduardo E. Alberto, Michael Ascnher, Diogo Onofre Gomes de Souza, Debora Guerini de Souza, João Batista Teixeira da Rocha

**Affiliations:** 1 Programa de Pós-Graduação em Ciências Biológicas: Bioquímica, ICBS, Universidade Federal do Rio Grande do Sul (UFRGS), Porto Alegre, Rio Grande do Sul, Brazil; 2 Departamento de Bioquímica e Biologia Molecular, Universidade Federal de Santa Maria (UFSM), Santa Maria, Rio Grande do Sul, Brazil; 3 Departamento de Biomedicina e Núcleo Comum de Saúde, Universidade Regional do Noroeste do Estado do Rio Grande do Sul (UNIJUÍ), Ijuí, Rio Grande do Sul, Brazil; 4 Departamento de Química, Universidade Federal de Minas Gerais—UFMG, Belo Horizonte, Minas Gerais, Brazil; 5 Departamento de Bioquímica, Instituto de Ciências Basicas da Saúde, Universidade Federal do Rio Grande do Sul (UFRGS), Porto Alegre, Rio Grande do Sul, Brazil; 6 Instituto Federal de Educação, Ciência e Tecnologia Sul-Rio-grandense (IFSul), Bagé, Rio Grande do Sul, Brazil; 7 Dipartimento di Scienze Chimiche, Università di Padova, Padova, Italy; 8 Department of Molecular Pharmacology, Albert Einstein College of Medicine, Bronx, New York, USA

## Abstract

Selenium (Se) is an essential micronutrient that participates in redox regulation and cellular homeostasis. Selenium-containing ionic liquids (ILsSe) have emerged as hybrid compounds combining the properties of organoselenium molecules with the tunable features of ionic liquids. In this study, we evaluated the cytotoxicity, interaction with mercury (Hg^2+^), pharmacokinetic profile, and antiproliferative activity of three ILsSe (C1, C2, and C3). Human peripheral blood mononuclear cells (PBMCs) were used to assess cytotoxicity, showing that ILsSe were generally well tolerated at low micromolar concentrations, although their effects were dependent on exposure time and dose. The interaction of ILsSe with Hg^2+^ was investigated using a thiol oxidase assay, with diphenyl diselenide (DPDS) as a reference compound. The results indicate that ILsSe can interact with Hg^2+^, likely through the formation of Se–Hg species. In parallel, in silico absorption, distribution, metabolism, excretion, and toxicity (ADMET) analysis suggested that these compounds have physicochemical properties compatible with central nervous system exposure, including predicted blood–brain barrier permeability. In U87-MG glioblastoma cells, compound C3 exhibited concentration and time-dependent antiproliferative effects at low micromolar concentrations. These effects were associated with alterations in redox homeostasis, modulation of cell cycle progression, and induction of late-stage cell death. In general, ILsSe are biologically active compounds whose effects depend on dose and exposure time, warranting further investigation to clarify their toxicological profile and mechanisms of action. This study highlights the importance and biological potential of selenium-containing compounds.

## 1 ∣ Introduction

Selenium (Se) is an essential trace element for humans and other living organisms, incorporated as selenocysteine into at least 25 selenoproteins involved in key physiological processes, including antioxidant defense, thyroid hormone metabolism, and redox homeostasis (Hariharan and Dharmaraj 2020; Shahidin et al. 2025; Labunskyy et al. 2014).

Organic Se compounds have attracted considerable attention due to their biological activities, among which are redox modulation, antiproliferative effects, and their ability to interact with electrophilic mercury species (Barbosa et al. 2017, 2024; de Freitas et al. 2009; Harmanci et al. 2017; Pablo Andrei Nogara et al. 2021; Nogueira et al. 2021; Radomska et al. 2021; Tinggi and Perkins 2022; Shahidin et al. 2025).

Ionic liquids (ILs) are salts composed of organic cations and organic or inorganic anions, typically with melting points below 100°C, whose physicochemical properties have enabled applications in electrochemistry, biochemistry, and pharmaceutical development (Beaven et al. 2024). In addition, ILs have been reported to exhibit biological activities, including antimicrobial and antitumor effects (Novello et al. 2024).

The combination of these features with Se bioactivity has led to the development of Se-containing ionic liquids (ILsSe) (Alberto et al. 2011; Klauke et al. 2018). However, despite their potential, information regarding their safety profile and biological effects remains limited (Che et al. 2025; Neves et al. 2023; Zhang et al. 2023).

In this context, the present study aimed to evaluate the biological effects of water-soluble ILsSe (Alberto et al. 2012). Specifically, we investigated their cytotoxicity in human peripheral blood mononuclear cells (PBMCs), a well-established model for toxicity assessment (He et al. 2004; de Souza Prestes et al. 2023), as well as their interaction with Hg^2+^. In addition, we assessed their antiproliferative activity in U87-MG human glioblastoma cells and performed in silico absorption, distribution, metabolism, and excretion (ADMET) analyses to provide an initial evaluation of their pharmacokinetic and toxicological properties.

## 2 ∣ Material and Methods

### 2.1 ∣ Chemicals and Reagents

**FIGURE 1 ∣:**
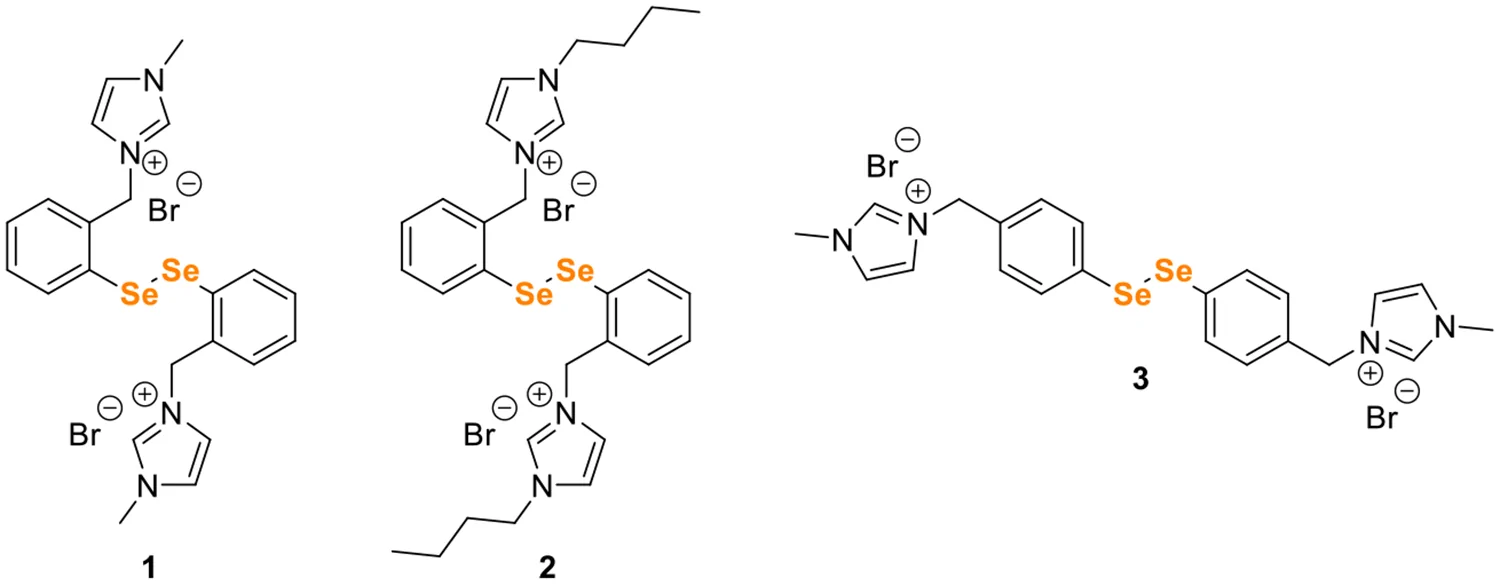
Chemical structure of the selenium compounds **1 (C1), 2 (C2), and 3 (C3)**.

Fetal bovine serum (FBS) and RPMI medium were obtained from Vitrocell Embriolife (Campinas, SP, Brazil). Antibiotic-antimycotic (100×) was obtained from Gibco, by Life Technologies (Grand Island, NY, USA). All other chemicals were obtained from Sigma-Aldrich (St. Louis, MO, USA). The ILsSe liquids **1** and **3** (Figure 1) evaluated in this work were synthesized and provided by Professor Eduardo Eliezer Alberto, from the Chemistry Department of the Universidade Federal de Minas Gerais (UFMG). The synthesis of compounds **1** (C1) and **3** (C3) evaluated in this work has been previously reported (Alberto et al. 2012).

Procedure for the synthesis of **2** (C2): A one-neck, round-bottomed flask was charged with alcohol **4** (0.186 mg, 0.5 mmol), acetic acid (5 mL), and 48% hydrobromic acid (3 mL). The resulting mixture was heated at 90°C for 3.5 h and then cooled to ambient temperature. Aqueous Na_2_CO_3_ was added to neutralize the reaction mixture, which was then extracted with Et_2_O (3 × 20 mL). The combined organic extracts were washed with brine, dried over MgSO_4_, filtered through Celite, concentrated, and dried under vacuum. Crude product **5** was used in the next reaction without further purification.

**SCHEME 1 ∣:**
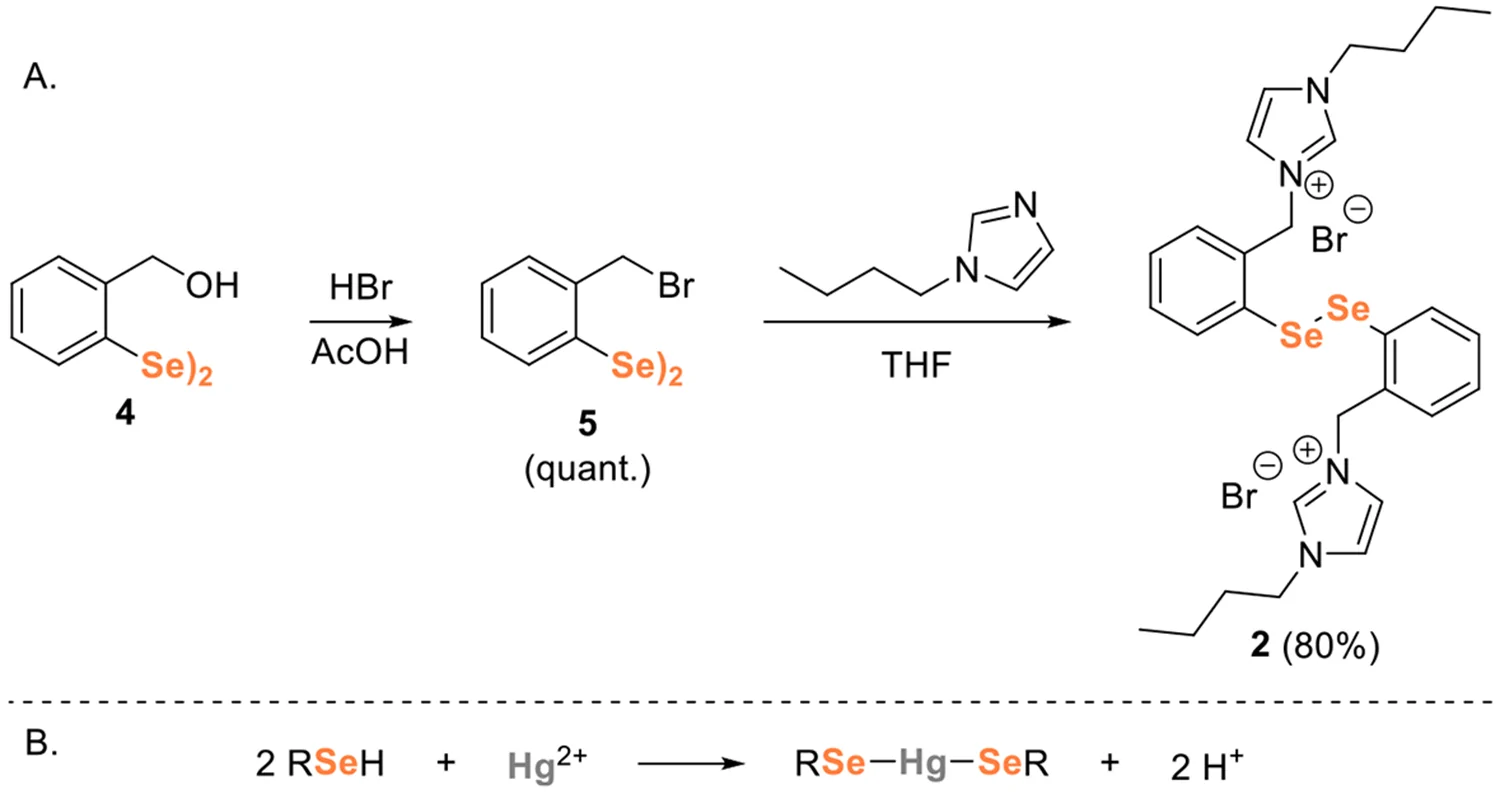
(A) Reaction sequence for the synthesis of selenium-containing ionic liquid 2. (B) Reaction between Se and Hg^2+^, forming a stable complex.

The tetrahydrofuran (THF, 2.5 mL) was added to the flask containing **5**, followed by 1-butylimidazole (0.26 mL, 1.1 mmol). The resulting solution was stirred at ambient temperature. Within 20 min of the addition of the imidazole, the formation of an orange oil could be observed. After 4 h of reaction, Et_2_O (10 mL) was added, and the resulting mixture was stirred for an additional 10 min. The oil in the reaction mixture was allowed to settle, and the organic layer was decanted. The supernatant solvent was removed, and an additional 10 mL of Et_2_O was added, repeating the previous steps a total of three times. After drying the orange oil under vacuum, 297 mg (80% yield) of the diselenide **2** was obtained (Scheme 1A).

Characterization data for C2: ^1^H NMR (CDCl_3_, 400 MHz) δ = 10.25 (s, 1H); 7.64 (s, 1H); 7.58–7.52 (m, 2H); 7.46 (s, 1H); 7.42–7.38 (m, 1H); 7.28–7.24 (m, 1H); 5.67 (s, 2H); 4.35 (t, *J* = 7.4 Hz, 2H); 1.92–1.84 (m, 2H); 1.40–1.31 (m, 2H); 0.92 (t, *J* = 7.3 Hz, 3H). ^13^C NMR (CDCl_3_, 100 MHz) δ = 137.5, 136.8, 136.1, 131.3, 130.7, 130.6, 130.3, 122.7, 122.4, 52.9, 49.8, 32.1, 19.4, 13.5; ^77^Se NMR (CDCl_3_, 76 MHz) δ = 459.7. HRMS (ESI, positive mode) calculated for C_14_H_18_N_2_Se [M]+: 294.0630, found 294.0632.

### 2.2 ∣ Human Subjects and Blood Collection

Peripheral blood was collected from healthy volunteers (three men, three women, mean age of 30 ± 10 years). Exclusion criteria included alcohol abuse or drug dependence, diagnosis of acute or chronic diseases, and any drug treatment for a period of 15 days. The blood collection protocol was conducted according to the principles expressed in the Declaration of Helsinki. The study was approved by the Human Research Ethics Committee (CAAE 67825122.1.0000.5346). All participants provided informed consent, and samples were coded and anonymized to ensure confidentiality.

### 2.3 ∣ PBMCs Isolation and Treatments

PBMCs were isolated following the methodology described by Böyum (1968), with some modifications. The peripheral blood (20 mL) was collected from each volunteer under aseptic conditions into tubes coated with sodium heparin (Hipolabor Farmacêutica Ltda, Belo Horizonte, MG, Brazil). Whole blood was diluted 1:1 with PBS buffer (136-mM NaCl, 2.68-mM KCl, 1.47-mM KH_2_PO_4_, 8.1-mM Na_2_HPO_4_, and pH 7.4) and layered over Ficoll-Paque Plus (GE Healthcare, Piscataway, NJ, USA) before centrifugation at 577 × *g* for 30 min to separate PBMCs. Cells were washed three times with PBS, followed by sequential centrifugation steps at 400 × *g* for 10 min, 256 × *g* for 10 min, and 178 × *g* for 5 min. Cells were then cultured in RPMI media supplemented with 10% FBS, 1% antibiotic-antimycotic, in the presence of the compounds under investigation at concentrations ranging from 1 to 200 μM and mercury chloride (HgCl_2_) from 5 to 100 μM, for 24, 48, and 72 h at 37°C and 5% CO_2_. Water was used as a vehicle. PBMCs were seeded at 1.25 × 10^6^ PBMCs/mL ELISA microplates for each experimental group.

Mercury experiments were conducted in accordance with institutional safety regulations in a biosafety level 2 laboratory using appropriate containment systems and personal protective equipment. Mercury waste was collected in labeled hazardous waste containers and disposed of by an authorized institutional service in accordance with environmental and occupational safety regulations.

### 2.4 ∣ U87-MG Cell Culture and Treatments

Human glioblastoma cells were purchased from the Rio de Janeiro Cell Bank (BCRJ) at passage 139. For assays, cells were cultured between passage 142–146 in DMEM (Dulbecco's Modified Eagle's medium; Gibco) containing 1 g/L of D-glucose, L-glutamine, 110-mg/mL sodium pyruvate and supplemented with 10% heat inactivated (30 min, 56°C) FBS (Gibco) and 1% penicillin/streptomycin (Gibco) in a humidified incubator at 37°C and 5% CO_2_. *Mycoplasma* contamination was assessed by PCR-based detection, following the protocol described by Uphoff and Drexler (2014). Molecular analyses were performed at the Multipurpose Laboratory of Molecular Biology (Institute of Basic Health Sciences, Federal University of Rio Grande do Sul, Brazil). No evidence of *Mycoplasma* contamination was detected in the cell cultures used for the experiments. The concentration used in this study was determined based on half-maximal inhibitory concentration (IC_50_) values obtained from concentration–response curves using the MTT reduction assay at three exposure times (24, 48, and 72 h). Among these, the 72-h time point was selected for subsequent mechanistic experiments because it showed the most pronounced biological response.

It is well established that IC_50_ values derived from the MTT assay may be influenced by changes in cellular redox state and mitochondrial metabolic activity, as tetrazolium reduction depends on metabolically active cells (Berridge et al. 2005). Therefore, to provide a complementary assessment of cytotoxicity, the IC_50_ was also determined at 72 h using the Trypan Blue exclusion assay.

The IC_50_ obtained by Trypan Blue was 2.4 μM, whereas the IC_50_ determined by MTT at the same time point was 11.27 μM. This discrepancy is expected, as the assays evaluate distinct biological parameters. Trypan Blue staining reflects loss of plasma membrane integrity and thus irreversible cell death (Strober 2015), while the MTT assay measures mitochondrial metabolic activity through the reduction of tetrazolium salts by cellular dehydrogenases (Stockert et al. 2012).

Considering that the aim of the subsequent experiments was to investigate metabolic and biochemical responses, an intermediate concentration (IC_50_-Int) between the mean of two IC_50_ values was selected. Therefore, a concentration of approximately 6.8 μM was used in the mechanistic experiments at 72 h of exposure. This approach enables the detection of molecular and metabolic alterations while preserving sufficient cell viability, thereby minimizing confounding effects associated with extensive cell death.

For all concentrations evaluated, stock solutions were prepared in advance to avoid alterations in the composition of the culture medium (DMEM). The treatment concentrations were standardized, and the vehicle control group (type I ultrapure water) was maintained at a constant proportion of 0.2% of the final volume in each well. In subsequent experiments using the intermediate concentration, the vehicle group was used as the control condition, hereafter referred to as Ctrl (H_2_O).

### 2.5 ∣ Cell Viability by Trypan Blue Method

The cell viability was determined using the Trypan Blue exclusion method, which assumes that nonviable cells are stained blue (Mishell and Shiigi 1980). Briefly, 10 μL of the 0.4% Trypan Blue reagent was added to 10 μL of PBMC suspension treated with 10 to 200 μM of the target compounds (C1, C2, and C3) for 24, 48, or 72 h. From the mixture, a 10-μL aliquot was placed in a Neubauer chamber, and the viable and nonviable cells were counted microscopically. After, concentration–response curves using lower concentrations (1, 5, and 10 μM) were performed at 24 and 72 h. Additionally, the potential interaction between 5 μM of the compounds and 10 μM of HgCl_2_ was evaluated after the same time of incubation. Cell viability was expressed as the percentage of viable cells.

### 2.6 ∣ Dehydrogenase Activity by MTT Assay

Determination of cellular toxicity of the compounds in PBMCS and U87-MG was also performed by MTT assay, following the methodology described by Mosmann (1983), with some modifications. Glioblastoma cells (5 × 10^3^) were seeded in 96-well plates (Kasvi) and incubated at 37°C for 48 h to allow adequate adhesion to the plates (confluence between 70% and 90%) according to Bramatti et al. (2024), Cwiklowska et al. (2018), and Rieder et al. (2026), with minor modifications. The method is based on the enzymatic reduction of MTT (3-(4,5-dimethylthia zol2-yl)-2,5-diphenyl tetrazolium bromide) to formazan crystals by intracellular dehydrogenases. In addition, PBMCs (1.25 × 10^6^ PBMCs/mL per group) were exposed to the compounds at concentrations of 1 to 200 μM, and the U87-MG cells were exposed to the concentrations of 1, 5, 10, and 50 μM (dissolved in type I ultrapure water), both for 24, 48, and 72 h, under the conditions described before. The potential interaction between 5 μM of the compounds and 10 μM of HgCl_2_ was also evaluated in PBMCs. Hydrogen peroxide (H_2_O_2_) at a final concentration of 1 mM was used as a positive toxicity control in the experiments with U87-MG cells. After treatment, MTT was added to the medium, and the samples were incubated for 4 h at 37°C, protected from light. Subsequently, the samples were centrifuged, the supernatant was discarded, and the formazan crystals were solubilized with DMSO p.a. After, the samples were readied at 540 nm in a SpectraMax M5 reader. Results were expressed as % of the control.

### 2.7 ∣ Thiol Oxidase Assay: Colorimetric Assay

The thiol oxidase assay was assessed according to the method previously described by Nogara et al. (2023). The ability of a thiol to reduce the Se–Se bond was measured indirectly by the Ellman method (Nogara et al. 2023; Ellman et al. 1961). The reaction medium (5% water) containing 1-mM dithiothreitol (DTT; a thiol source), 50 μM (PhSe)_2_, 50 μM Compound C3, and 50-μM mercury chloride (HgCl2) was incubated at 37°C for 24 h. Aliquots (20 μL) were collected at 0, 1, 2, 3, 4, 5, 6, and 24 h for the colorimetric reaction. In the colorimetric assay, aliquots were mixed with 180 μL of 2-mM 5,5′-dithiobis(2-nitrobenzoic acid) (DTNB; chromophore agent), and the absorbance was measured spectrophotometrically at 412 nm.

### 2.8 ∣ Biochemical Assays

#### 2.8.1 ∣ Preparation of Total Cell Lysates

Total U87-MG cell lysis was performed according to the method described by Rieder et al. (2026), Bramatti et al. (2024), and Pires et al. (2022), with some minor modifications. Briefly, cells were grown in 6-well plates (Kasvi) until they reached 80%–90% confluence. After the expected confluence, we removed the old medium and added a new medium with the treatments. Based on previous assays, we decided to continue with only the following groups for subsequent assays: (1) control—water vehicle, (2) C3 in IC_50_-Int (6.8 μM). After 72 h of exposure, the treatment media were removed, and the cells were washed with 1× phosphate-buffered saline (PBS). After washing, to transfer the cells to microtubes, trypsin was added for 5 min to detach the cells, and inactivated with DMEM (10% FBS and 1% penstrep). Then, the contents obtained from each well were transferred to a microtube and centrifuged at 1000 × *g* for 5 min at 4°C. Subsequently, the supernatant was discarded, and the cell pellet was exposed for 10 min to a Triton solution (0.05%). The samples were then lysed by moving them “up and down” in a 1-mL insulin syringe. The samples were then centrifuged (1000 × *g* for 10 min at 4°C), and the supernatant was separated for protein measurement and biochemical tests. The protein content of the homogenate was determined using a Thermo Scientific NanoDrop Lite and normalized to ensure equal protein loading across all conditions prior to the enzymatic assay.

#### 2.8.2 ∣ Determination of the Total Thiol Content

The total thiol level was determined according to the method previously described by Ellman et al. (1961). The reaction system consisted of 170 μL of 0.1-M KPB (pH 7.4), 20 μL of sample, and 10 μL of 10-mM DTNB. Blank samples were prepared by adding the same amount of tissue in the absence of DTNB. After 60 min of incubation at 37°C in the dark, absorbance was measured at 412 nm using a SpectraMax M5 reader. The reaction of DTNB with the thiol groups was analyzed by measuring the mean absorbance minus blank. Results were expressed as % of the control.

#### 2.8.3 ∣ Glutathione Peroxidase Activity

The glutathione peroxidase activity (GPx activity) was determined according to the method previously described by Sattar et al. (2024), with some minor modifications. The reaction system consisted of 180 μL of 0.1-M KPB (pH 7.4), 10 μL of 10-mM EDTA, 10 μL of 10-mM Azide, 5 μL of 10-mM Tertiary-butyl hydroperoxide (TBuOOH), and 10 μL of the sample. After preparing the *KBP-EDTA-azide-tBuOOH* system, we added 5 μL of 10-mM GSH and incubated it for 180 min at 37°C. After incubation, 16.25 μL of HNa_2_OP4 (80 mg/mL) was added, followed by 20 μL of 20-mM DTNB. The absorbance was measured at 412 nm using a SpectraMax M5 reader. The reaction of DTNB was analyzed by mean absorbance minus blank. Results were expressed as % of the control.

### 2.9 ∣ Measurement of Reactive Oxygen Species (ROS) Generation

ROS production was measured using DCFH-DA. Cells were incubated with 5-μM DCFH-DA for 30 min at 37°C in the dark. After staining, cells were washed twice with PBS and immediately analyzed by flow cytometry. Data acquisition was performed using a BD Accuri C6 flow cytometer (BD Biosciences). Viable cells were initially identified based on forward scatter (FSC) versus side scatter (SSC) parameters. Doublets were excluded using FSC-A versus FSC-H plots. For each sample, 10,000 events were acquired in the viable cells gate. Unstained cells were used as negative controls for gating.

### 2.10 ∣ Annexin V-FITC/Propidium Iodide (PI) and Cell Cycle Analysis

To evaluate cell death and cell cycle, 1.5 × 10^5^ U87-MG cells/well were cultured in 6-well plates. After 48 h, cells were treated with Int-Conc of C3 (6.8 μM) for 72 h, under standard cell culture conditions (37°C, 5% CO_2_). Apoptotic and/or necrotic cells were quantified with Annexin V FITC/PI double staining kit following the manufacturer's guidelines (BD Biosciences, San Diego, USA, cat#556547). The U87-MG cells (10^5^ cells/mL) were suspended in a buffer comprising Annexin V-FITC and PI for 15 min. For cell cycle analysis, U87-MG cells (10^6^ cells/mL) were suspended in 300-μL staining solution containing Tris–HCl (0.5 mM, pH 7.6), trisodium citrate (3.5 mM), Nonidet P 40 (0.1% v/v), RNase (100 μg/mL, cat#R6513, Sigma-Aldrich, Burlington, Massachusetts, USA) and PI (50 μg/mL, cat#P4170 Sigma-Aldrich, Burlington, Massachusetts, USA), for 15 min in the dark. Data were acquired by a flow cytometer (BD Accuri BD Biosciences, USA), and analyzed using FlowJo v10.9.0 software (BD Biosciences, New Jersey, USA).

For apoptotic cell death, cells were classified as follows: viable cells (Annexin V and PI negative), early apoptotic cells (Annexin V positive and PI negative), and late apoptotic or necrotic cells (Annexin V and PI positive or Annexin V negative and PI positive).

### 2.11 ∣ ADMET Simulation Analysis

The pkCSM pharmacokinetic was used to predict the druggability of the compounds based on Lipinski's rule of five and different pharmacokinetic classes (Daina et al. 2017; Kappenberg et al. 2023; Pires et al. 2015). ADMET properties were predicted using the pkCSM webserver based on the SMILES representation of compounds C1, C2, and C3 (including their counterions), DPDS, and imidazole (1-butylimidazole). The analysis was performed using the pkCSM platform (accessed in September 2025).

### 2.12 ∣ Statistical Analysis

Statistical analyses were performed using GraphPad Prism version 8.00 for Windows (GraphPad Software, La Jolla, CA, USA). Data are presented as mean ± SD of 3–4 independent biological experiments. Data normality was assessed using the Shapiro–Wilk test, and homogeneity of variances was verified before applying parametric tests. For comparisons involving two groups, an unpaired two-tailed Student's *t*-test was used. For multiple group comparisons, one- or two-way ANOVA followed by Tukey's post hoc test was applied, as appropriate. Differences were considered statistically significant when *p* < 0.05. Exact *p*-values are reported for statistically significant differences.

## 3 ∣ Results

### 3.1 ∣ Influence of ILs Containing Se on the Cell Viability of PBMCs

**FIGURE 2 ∣:**
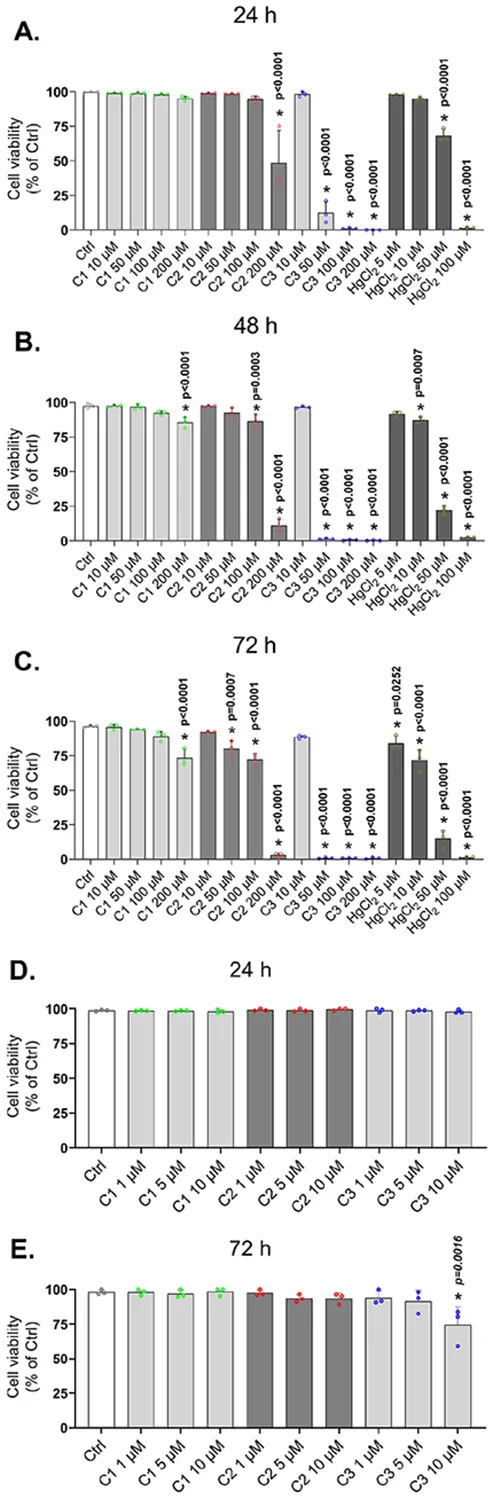
Cell viability of PBMCs assessed by the Trypan Blue method. To determine the IC_50_ values, concentration–response curves were first generated for ILsSe and HgCl_2_. Results are expressed as mean ± standard deviation (SD) of the percentage of viable cells after 24 (A), 48 (B), and 72 h (C) of exposure to the compounds at 10, 50, 100, and 200 μM, and mercury at 5, 10, 50, and 100. Subsequently, additional concentration–response curves using lower doses (1, 5, and 10 μM) were performed at 24 (D) and 72 h (E). Statistical differences were assessed by one-way ANOVA followed by Tukey's post hoc test. All comparisons shown in the graph are relative to the respective control group (Ctrl), and exact *p-*values for significant differences are reported in the figure. Colored circles indicate the biological variability among donors within each group.

Firstly, concentration–response curves were generated for Se-containing ionic compounds at 10, 50, 100, and 200 μM, as well as for HgCl_2_ at 5, 10, 50, and 100 μM, across different time points (24, 48, and 72 h) (Figure 2A-C), to determine their respective IC_50_ values (Table 1). Subsequently, the effects of compounds C1, C2, and C3 at lower concentrations (1, 5, and 10 μM) on PBMCs viability were analyzed after 24 and 72 h of exposure using the Trypan Blue exclusion assay (Figure 2D,E). After 24 h (Figure 2D), no significant differences in cell viability were observed between treated groups and the control, with all conditions maintaining viability close to 100%, indicating the absence of acute cytotoxic effects at the tested concentrations. After 72 h (Figure 2E), most treatments still preserved high cell viability, similar to the control group (Ctrl). However, exposure to compound C3 at 10 μM significantly reduced PBMCs viability compared with the control group (*p* = 0.0016), demonstrating a time- and concentration-dependent cytotoxic effect for this compound at the highest concentration tested. Overall, the results indicate that compounds C1 and C2 were well tolerated under all tested conditions, whereas C3 displayed moderate cytotoxicity only after prolonged exposure at higher concentrations.

### 3.2 ∣ ILsSe Differentially Affect Dehydrogenase Activity of PBMCs

**FIGURE 3 ∣:**
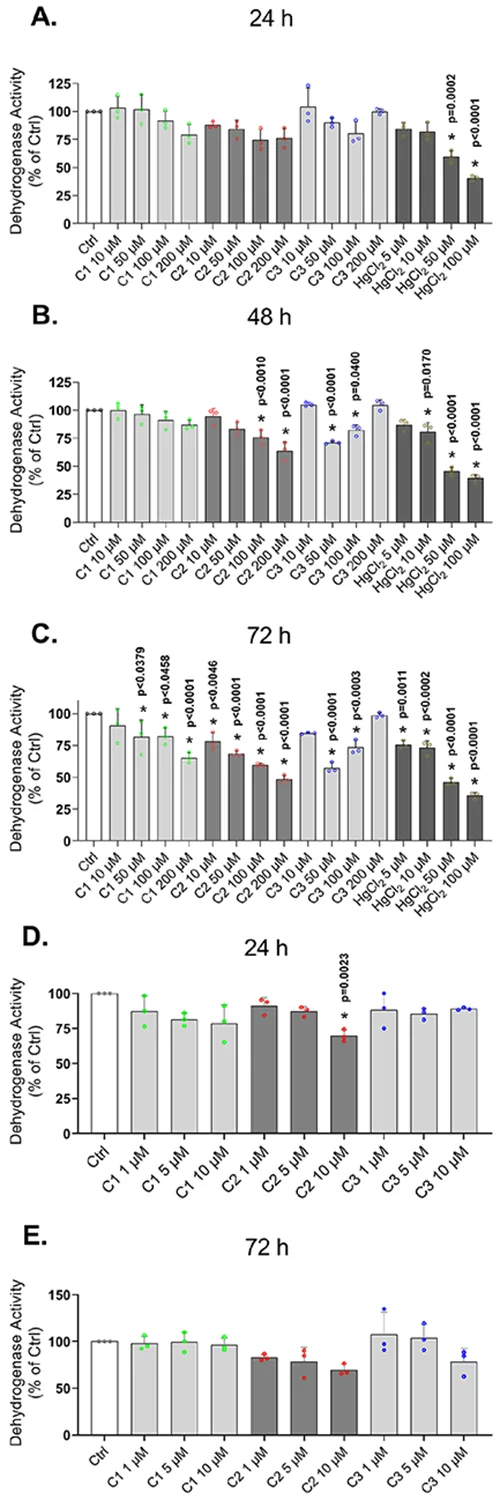
Intracellular dehydrogenase activity of PBMCs by MTT assay. Concentration–response curves were first performed for ILsSe and HgCl_2_. In this case, after 24 (A), 48 (B), and 72 h (C) of exposure to the compounds at 10, 50, 100, and 200 μM, and mercury at 5, 10, 50, and 100 μM, results are expressed as mean ± standard deviation (SD) of the percentage of viable cells. Subsequently, additional concentration–response curves using lower doses (1, 5, and 10 μM) were performed at 24 (D) and 72 h (E), and these results are expressed as mean ± standard deviation (SD) of the percentage of viable cells. Statistical differences were assessed by one-way ANOVA followed by Tukey's post hoc test. All comparisons shown in the graph are relative to the respective control group (Ctrl), and exact *p*-values for significant differences are reported in the figure. Colored circles indicate the biological variability among donors within each group.

Intracellular dehydrogenase activity in PBMCs showed time- and concentration-dependent effects after exposure to ILsSe (C1–C3) and HgCl_2_. At 24 h, ILsSe did not cause relevant effects. HgCl_2_ already reduced activity significantly at 50 and 100 μM (*p* = 0.002 and *p* < 0.0001, respectively). At 48 h, ILsSe induced decreases mainly at higher concentrations (≥ 50–200 μM), especially for C2 and C3. HgCl_2_ showed marked cytotoxicity starting at 10 μM (*p* = 0.0170), with stronger effects at 50 and 100 μM (*p* < 0.0001). At 72 h, ILsSe significantly reduced activity at intermediate to high concentrations, with stronger effects for C2 and C3. HgCl_2_ caused pronounced reductions at all concentrations tested (*p* ≤ 0.0011). At lower concentrations (1–10 μM), ILsSe had minimal effects at 24 h, except for C2 at 10 μM (*p* = 0.0023). After 72 h (Figure 3E), no significant differences were detected among the experimental groups. Overall, these findings indicate that compounds C1 and C3 were generally well tolerated, while C2 induced a transient reduction in PBMCs metabolic activity at the highest concentration after short-term exposure.

### 3.3 ∣ ILsSe Do Not Protect Against Hg^2+^ Toxicity in PBMCs

**FIGURE 4 ∣:**
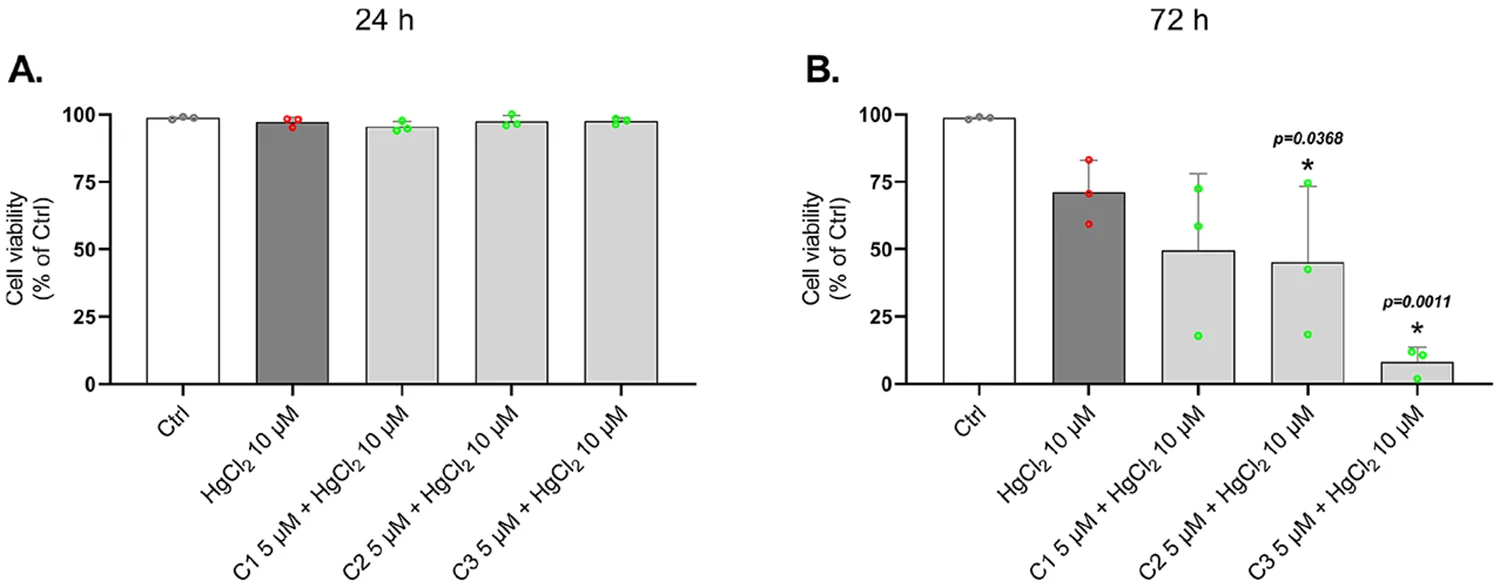
Evaluation of the interaction of 5-μM ionic liquids containing selenium with 10-μM mercury (Hg^2+^) toxicity in PBMCs by Trypan Blue method. The results are expressed as the mean ± SD of the percentage of viable cells of three independent experiments, after 24 (A) or 72 h (B) of exposure to the compounds. Statistical differences were assessed by one-way ANOVA followed by Tukey's post hoc test. All comparisons shown in the graph are relative to the control group (Ctrl), and exact *p*-values for significant differences are reported in the figure. The colored circles indicate the biological variability among each donor for each group.

After verifying the potential cytotoxicity induced by ILsSe in PBMCs, the interaction between these compounds (5 μM) and Hg^2+^ (10 μM) was evaluated. In the Trypan Blue assay (Figure 4), no significant changes were observed after 24 h (Figure 4A), with all groups maintaining values close to the control (Ctrl). After 72 h (Figure 4B), HgCl_2_ alone reduced PBMCs viability, whereas co-exposure with the ILsSe further modulated this effect. The combination of C2 (5 μM) with HgCl_2_ significantly decreased viability compared with the control (*p* = 0.0368), while the association of C3 (5 μM) with HgCl_2_ produced the most pronounced reduction in viability (*p* = 0.0011). These findings indicate that prolonged exposure enhanced mercury toxicity, particularly in the presence of C2 and C3.

**FIGURE 5 ∣:**
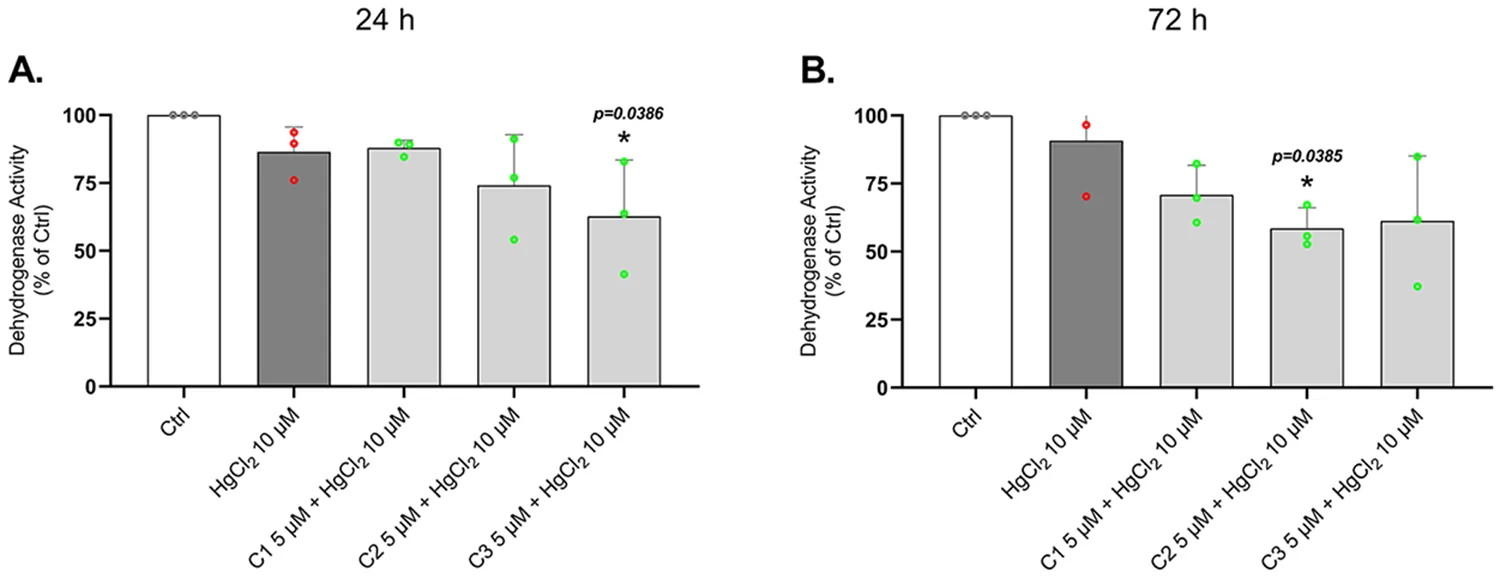
Evaluation of the interaction of 5-μM ionic liquids containing selenium with 10 μM mercury (Hg^2+^) in dehydrogenase activity of PBMCs by MTT method. The results are expressed as the mean + SD of the percentage of viable cells of three independent experiments, after 24 (A) or 72 h (B) of exposure to the compounds. The data were normalized to Ctrl and reported as a percentage of dehydrogenase activity. The results were expressed as the mean ± SD of three independent experiments by one-way ANOVA followed by Tukey's test. All comparisons shown in the graph are relative to the control group (Ctrl), and exact *p*-values for significant differences are reported in the figure. The colored circles indicate the biological variability among each donor for each group.

In the MTT assay (Figure 5), after 24 h (Figure 5A), most groups showed metabolic activity comparable to the control. However, the combination of C3 (5 μM) with HgCl_2_ significantly reduced intracellular dehydrogenase activity (*p* = 0.0386), suggesting an early impairment of mitochondrial/metabolic function. After 72 h (Figure 5B), a stronger effect was observed: co-exposure with C2 (5 μM) and HgCl_2_ decreased dehydrogenase activity relative to the control (*p* = 0.0385), while the remaining groups showed reduced values without statistical relevance. Overall, the data demonstrate that mercury toxicity became more evident after prolonged exposure and that its interaction with ILsSe was compound-dependent. Among the tested compounds, C2 and especially C3 potentiated the deleterious effects of Hg^2+^ on PBMCs viability and metabolic activity.

### 3.4 ∣ ILsSe and Diphenyl Diselenide Reduced Intermediates Interact With Hg^2+^ and Inactivate Their Thiol Oxidase Activity

**FIGURE 6 ∣:**
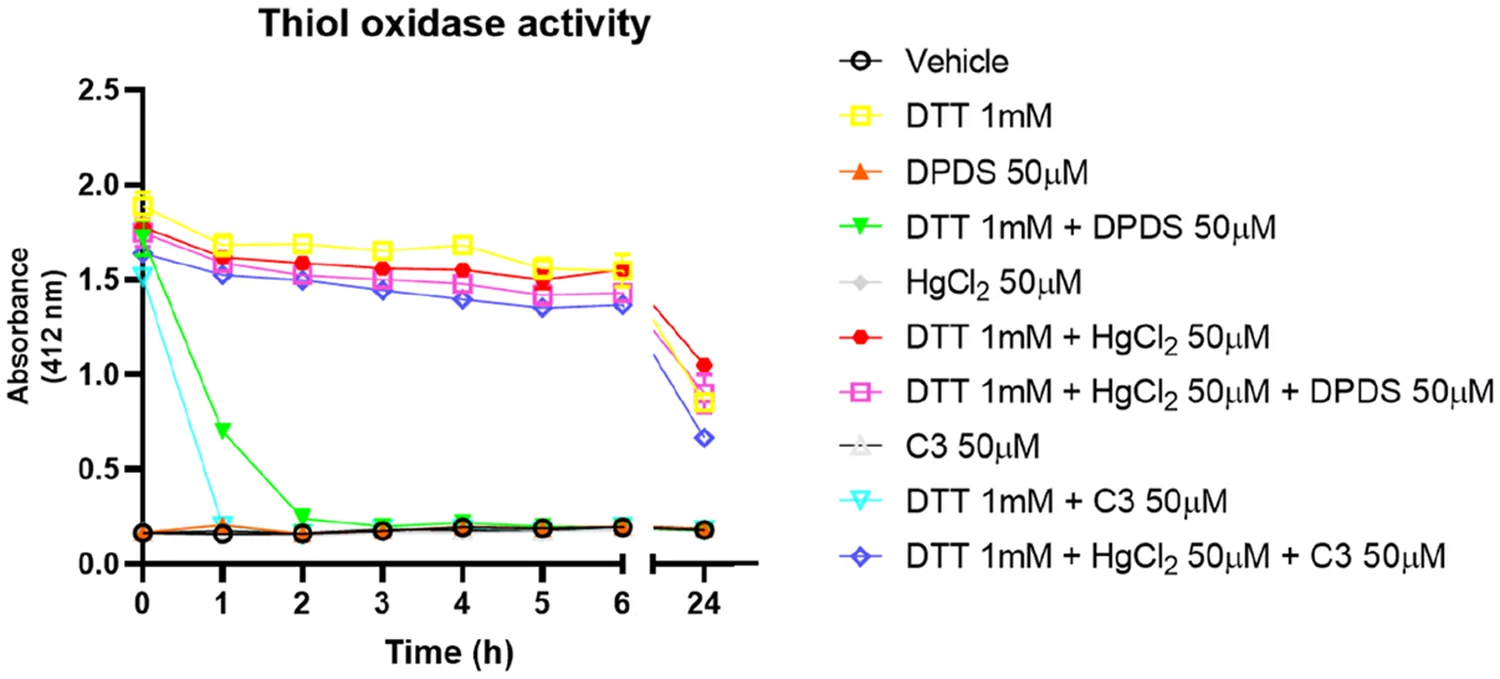
Thiol oxidase activity of C3, DPDS, and HgCl_2_. Time course of thiol oxidase activity measured as absorbance at 412 nm over 24 h. The group (dark blue) containing C3, HgCl_2_, and DTT demonstrates the ability of C3 to interact with HgCl_2_ and prevent DTT oxidation, whereas in the group (light blue) containing only C3 and DTT, oxidation of DTT occurs within the first few hours. Similar behavior was observed at 25 μM for C3, DPDS, and Hg species, although these data are not shown to avoid overloading the figure.

In the thiol oxidase assay (Figure 6), to verify whether reduced intermediates of ILsSe interacts with Hg^2+^ after its reduction to its selenol intermediate, diphenyl diselenide, (DPDS), an organoselenic compound well characterized in the literature for interacting with Hg^2+^, and compound C3 were used. Both compounds demonstrated interaction with Hg^2+^. Notably, no decrease in DTT absorbance was observed in the Hg^2+^ + DTT group, indicating that Hg^2+^ alone does not promote changes in DTT absorbance under these conditions. Similarly, in the cotreatment groups (C3 or DPDS + Hg^2+^ + DTT), the absorbance of DTT remained stable. In contrast, in the absence of Hg^2+^ (C3 or DPDS + DTT), a rapid decrease in DTT absorbance was observed within the first few hours, indicating a strong oxidation of DTT thiol groups. Taken together, these results suggest that ILsSe form a stable complex with Hg^2+^, since in the presence of DTT and Hg^2+^ there is no decrease in the absorbance of DTT.

### 3.5 ∣ ILs Containing Se Exhibit Cytotoxic Effects in Glioblastoma Cells

**FIGURE 7 ∣:**
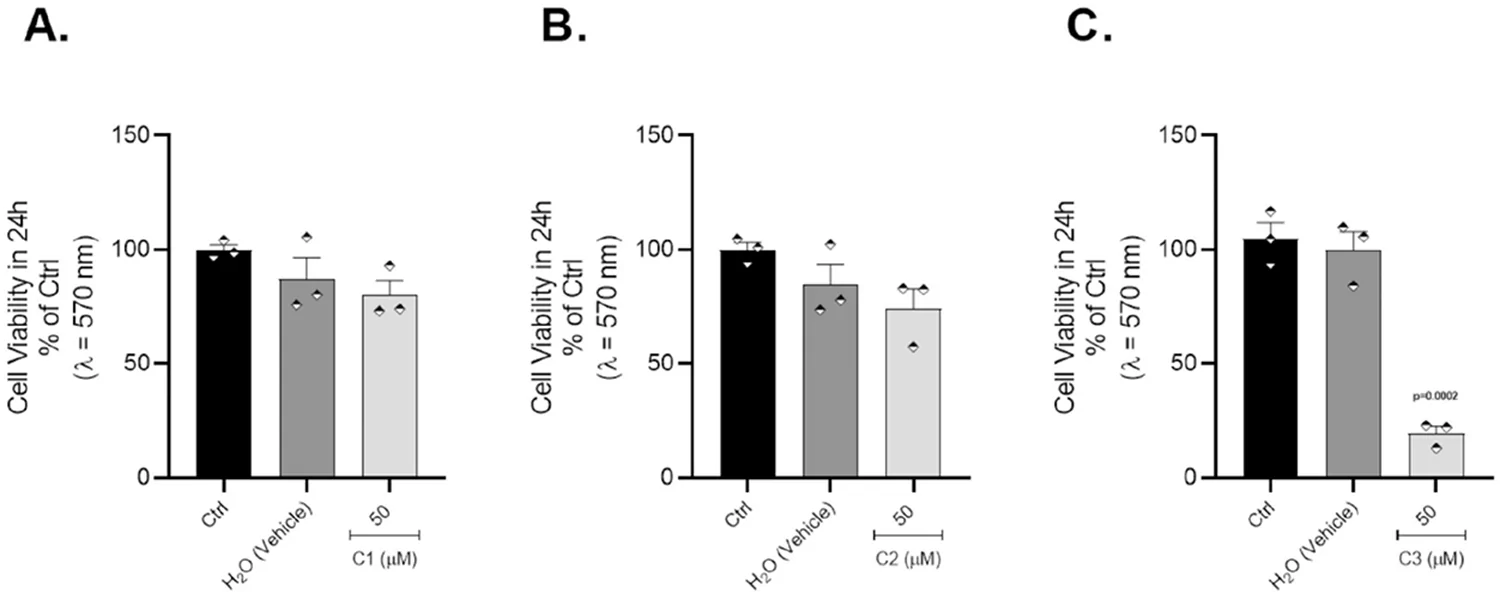
Evaluation of the citotoxicity capacity of ionic liquids containing selenium in human glioblastoma cells (U87-MG) by dehydrogenase activity using the MTT assay. U87-MG cells were exposed to the compounds for 24 h at a single concentration of 50 μM (A, B, and C). Only compound C3 showed the ability to reduce the cell volatility of glioblastoma cells at a concentration of 50 μM. The data were normalized to Ctrl and reported as a percentage. The results were expressed as the mean ± SD of dehydrogenase activity 24 h after exposure to ionic liquids containing Se. Statistical differences were assessed by one-way ANOVA followed by Tukey's post hoc test. All comparisons shown in the graph are relative to the vehicle control group, and exact *p*-values for significant differences are reported in the figure.

**FIGURE 8 ∣:**
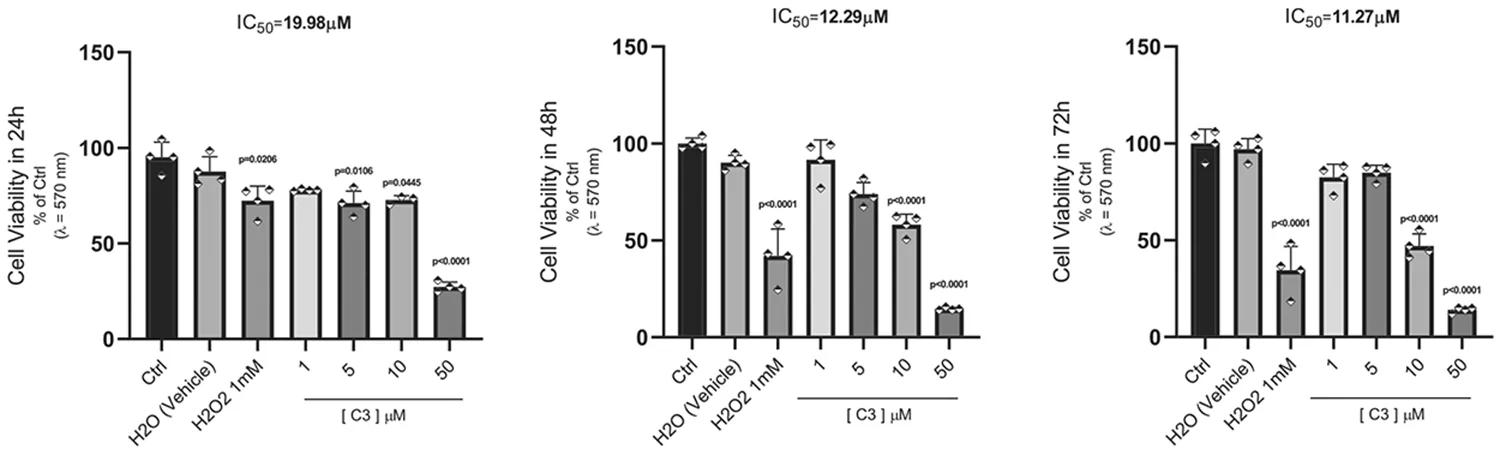
Cytotoxic effects of compound C3 in U87-MG cells. After identifying C3 as the only compound exhibiting cytotoxic activity at 50 μM, its potency was further evaluated using a concentration–response curve (1, 5, 10, and 50 μM) at different time points: (A) 24, (B) 48, and (C) 72 h. IC_50_ values were determined for each time point and showed a time-dependent decrease. Notably, C3 significantly reduced cell viability at 10 μM across all evaluated time points. Data are expressed as percentage of control (Ctrl) and presented as mean ± SD of independent biological replicates (*n* = 3–4). Statistical analyses were performed using one-way ANOVA followed by Tukey's post hoc test. All comparisons are relative to the vehicle control group (H_2_O), and exact *p*-values are indicated in the graphs.

**FIGURE 9 ∣:**
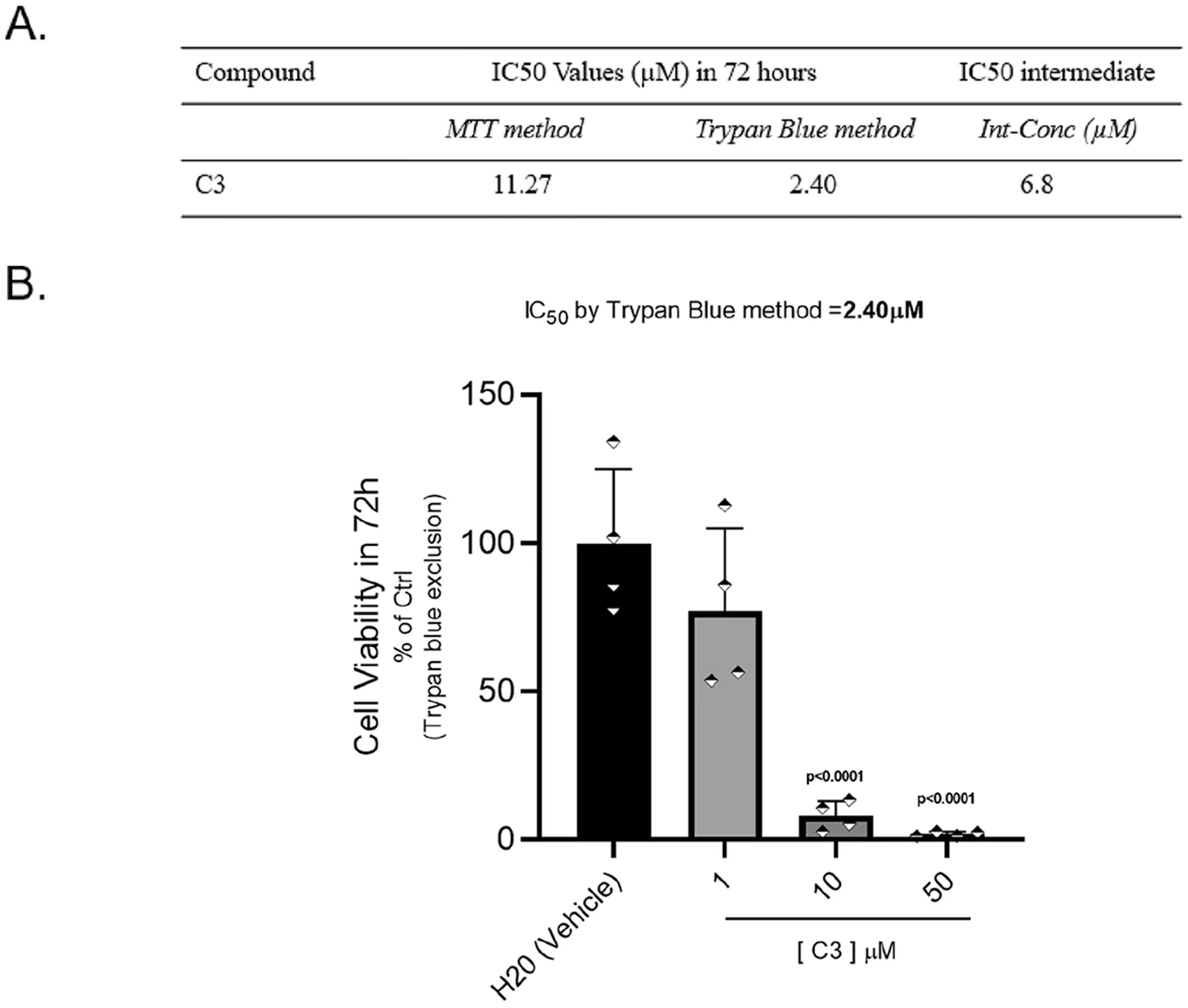
(A) Table with IC_50_ values (μM) of compound C3 in glioblastoma cells after 72 h of exposure, determined by MTT and Trypan Blue assays, with the corresponding intermediate concentration (6.8 μM). The table highlights the difference in IC_50_ values obtained by the two methods. Based on this discrepancy, the intermediate concentration was defined as the mean of the IC_50_ values from both assays. (B) Cell viability of glioblastoma (U87-MG) cells after 72 h of exposure to compound C3, assessed by the Trypan Blue exclusion assay. Cells were treated with increasing concentrations of C3 (1, 10, and 50 μM), and viability is expressed as percentage of control (Ctrl). The IC_50_ value determined by this method was 2.40 μM. Data are presented as mean ± SD of independent biological replicates (*n* = 3–4). Statistical analysis was performed using one-way ANOVA followed by Tukey's post hoc test. All comparisons are relative to the control group (H_2_O), and exact *p*-values are indicated in the graph, with a significant reduction in cell viability observed at higher concentrations.

After evaluating the toxicity and interaction with Hg^2+^ of ILsSe in PBMCs, their antiproliferative potential was assessed in human glioblastoma cells (U87-MG) using MTT and trypan blue viability assays. Among the evaluated compounds, we identified that C3 (Figure 7C), at a concentration of 50 μM, reduced the viability of tumor cells. Subsequently, we performed a concentration–response curve (1, 5, 10, and 50 μM) of C3 across three time points (24, 48, and 72 h, Figure 8A-C, respectively). The curves demonstrated that C3 still exhibits cytotoxicity capacity against U87-MG cells up to a concentration of 10 μM. The concentration used in the mechanistic experiments was defined based on IC_50_ values obtained by MTT and Trypan Blue assays after 72 h of exposure (Figure 9), the time point that showed the strongest biological response. Therefore, an intermediate concentration (6.8 μM) was selected for the subsequent investigations performed in glioblastoma cells.

### 3.6 ∣ ILsSe C3 Decreased Total Thiol Levels in Glioblastoma Cells

**FIGURE 10 ∣:**
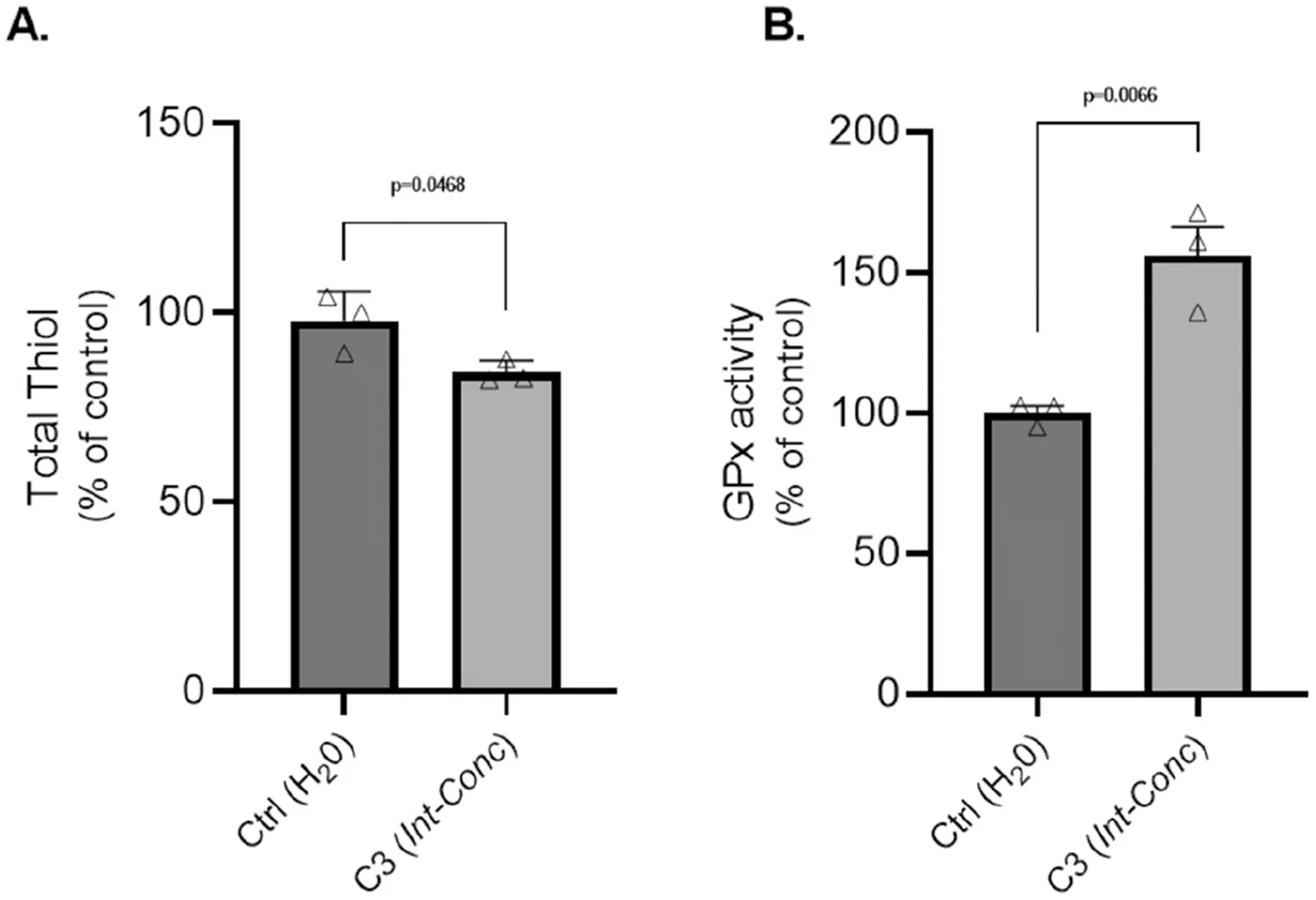
Effects of C3 on thiol content and glutathione peroxidase activity (GPx) in U87-MG cells after 72-h exposure. Cells were treated with C3 (6.8 μM) and compared to control (H_2_O). (A) Total thiol levels (% of control). (B) Glutathione peroxidase (GPx) activity (% of control). C3 treatment significantly reduced total thiol levels (*p* = 0.0468) while increasing GPx activity (*p* = 0.0066). Data are presented as mean ± SD of independent biological replicates (*n* = 3–4) and analyzed by unpaired Student's *t*-test. Exact *p*-values are indicated in the figure.

U87-MG cells treated with an intermediate concentration of C3 (6.8 μM) showed a significant reduction in total thiol content compared to the control group (*p* = 0.0468) (Figure 10A).

### 3.7 ∣ ILsSe C3 Increased GPx Activity in Glioblastoma Cells

U87-MG cells treated with an intermediate concentration of C3 (6.8 μM) showed a significant increase in GPx activity compared to the control group (*p* = 0.0066) (Figure 10B).

### 3.8 ∣ ILsSe C3 Exhibit Antioxidant Potential by Reducing ROS Levels in Glioblastoma Cells

**FIGURE 11 ∣:**
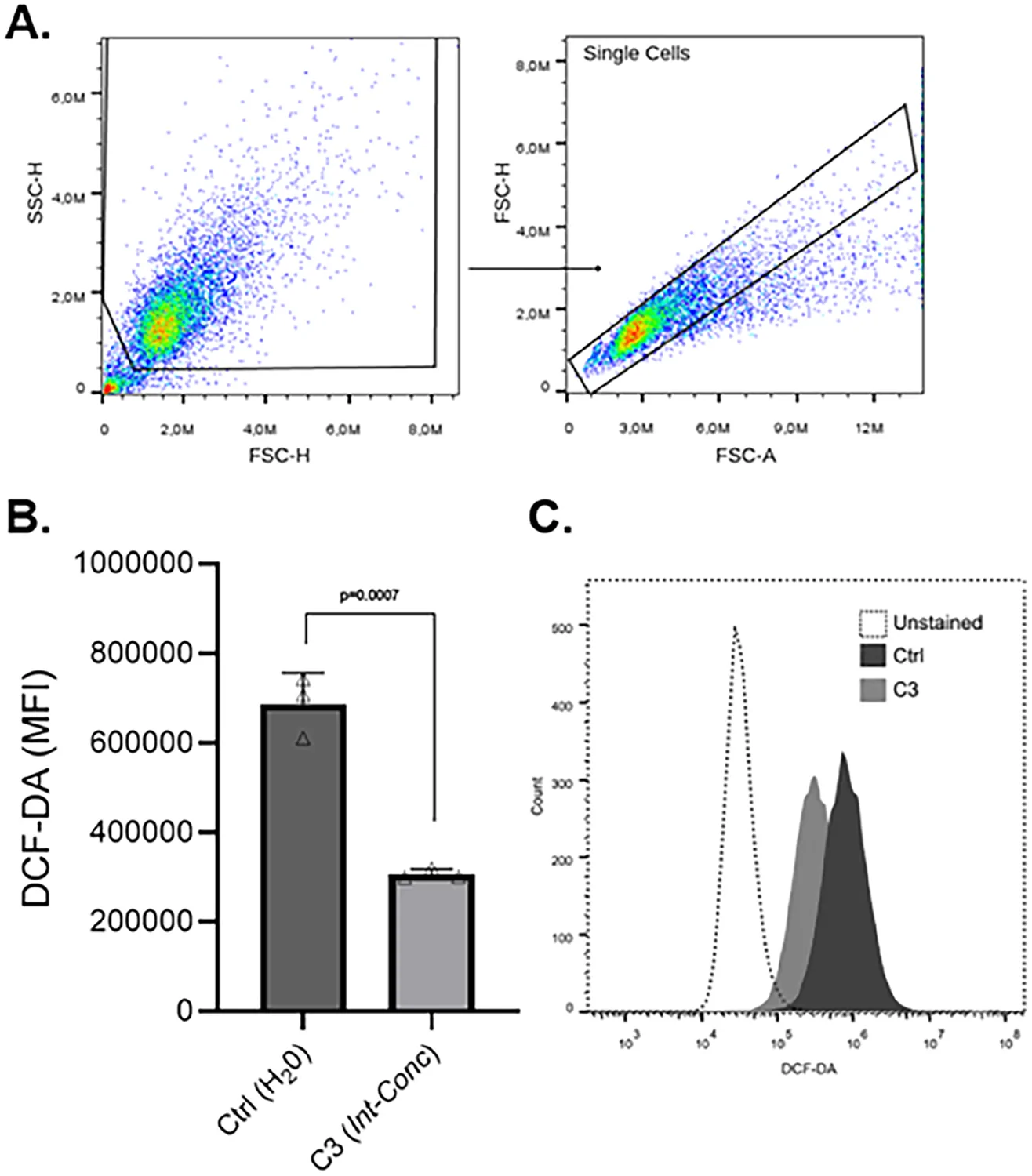
Effects of C3 on intracellular ROS levels in U87-MG cells after 72-h exposure. (A) Representative flow cytometry plots detailing the gating strategy. Debris was removed from the analysis based on forward and side scatter profiling (FSC × SSC). The gate was refined on single cells (FSC-A × FSC-H). Cells were treated with C3 (6.8 μM) and compared to the control (H_2_O). Intracellular ROS levels were assessed using the DCFH-DA assay and expressed as median fluorescence intensity (MFI). (B) C3 treatment significantly reduced ROS levels compared to the control group (*p* = 0.0007). (C) Representative histogram from flow cytometry. Data are presented as mean ± SD of independent biological replicates (*n* = 3–4) and analyzed by unpaired Student's *t*-test. Exact *p*-values are indicated in the figure.

U87-MG cells treated with an intermediate concentration of C3 (6.8 μM) showed a significant reduction in ROS production compared to the control group (*p* = 0.0007) (Figure 11A-C).

### 3.9 ∣ ILsSe C3 Induces G2/M Cell Cycle Arrest and Late Apoptotic Cell Death in U87-MG Glioblastoma Cells

**FIGURE 12 ∣:**
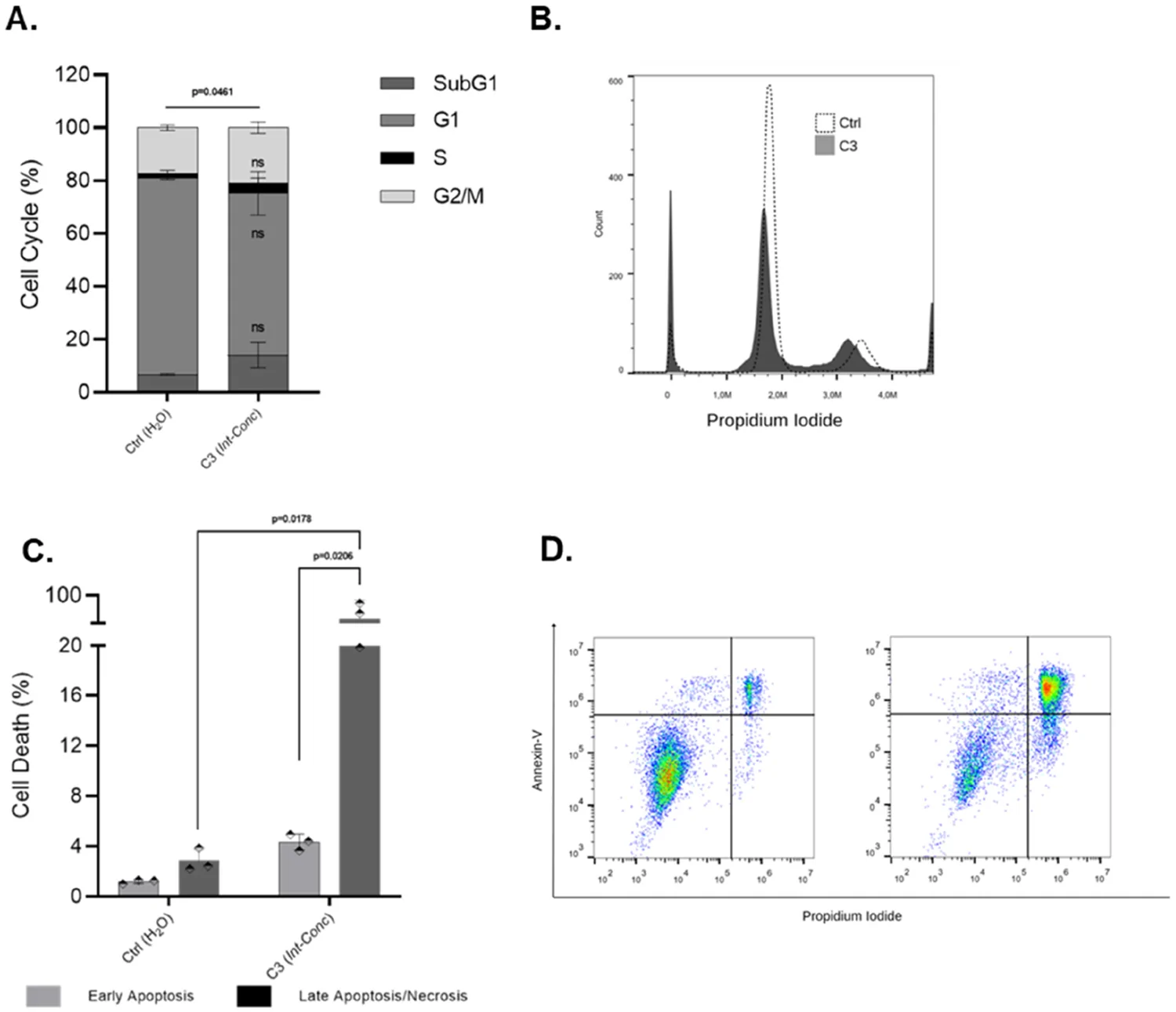
C3 modulates cell cycle progression and induces cell death in U87-MG cells after 72-h exposure. (A) Cell cycle of U87-MG cells after 72 h of treatment with C3 (6.8 μM). Analysis shows a significant increase in the SubG1 population compared to control (*p* = 0.0461), while no significant differences were observed in G1, S, and G2/M phases. (B) Representative histogram from flow cytometry. (C) Cell death was assessed by Annexin V-FITC/PI staining after 72 h of treatment and analyzed by flow cytometry. Quantification revealed a significant increase in total cell death in C3-treated cells (6.8 μM) compared to control (*p* = 0.0178), mainly driven by late apoptosis/necrosis. No significant differences were observed in early apoptosis between groups. (D) Dot plot illustrates the viable cells (lower left quadrant), early-phase apoptotic cells (upper left quadrant), and late-phase apoptotic/necrotic cells (lower and upper right quadrant). The left panel represents the control group, and the right panel represents C3-treated cells. Data are presented as mean ± SD of independent biological replicates (*n* = 3–4) and analyzed by Student's *t*-test analysis or by two-way ANOVA followed by Tukey's post hoc test. Exact *p*-values for significant differences are reported in the figure.

The compound C3 (6.8 μM) promoted cell cycle arrest in the G2/M phase (*p* = 0.0461) (Figure 12A,B) and increased late apoptosis/necrosis (*p* = 0.0178) in U87-MG cells, with no changes in early apoptosis (Figure 12C,D).

### 3.10 ∣ ADMET Simulation Analysis: ILs Containing Se Exhibit Similar ADMET Properties to DPDS

ADMET analysis was performed to evaluate the pharmacokinetic and toxicological properties of compounds C1, C2, C3, DPDS, and the imidazole group (Table 2). The prediction parameters were analyzed using pkCSM software (Pires et al. 2015). All compounds showed low water solubility, low skin permeability, and high intestinal absorption, and all tested positive for the substrate, including the P-glycoprotein inhibitor. The studied compounds showed high permeability values across the blood–brain barrier (BBB) and the central nervous system (CNS). Regarding cytochrome metabolism, they showed similar interactions with the respective isoforms evaluated here. Furthermore, positive values for the renal OCT2 substrate were observed. Additionally, the assessed compounds showed no toxicity in the AMES test or hepatotoxicity. The maximum tolerated dose (MTD) was low for all compounds. Finally, they were considered inhibitors of the hERG II potassium channel. Regarding the LD_50_ and LOAEL evaluation, all compounds presented similar values.

## 4 ∣ Discussion

Given the demand for innovative therapeutic strategies, particularly against aggressive glioblastomas (Teraiya et al. 2023; Weller et al. 2015), the evaluation of both the safety and biological potential of newly synthesized compounds is timely and essential. In this context, the present study investigated water-soluble ILsSe, a class of hybrid molecules that merge the chemical versatility of ILs with the well-recognized biological relevance of organoselenium derivatives. While organoselenium compounds have been extensively explored for their ability to mimic and enhance the functions of human selenoproteins, particularly in redox regulation and cytoprotection, they have also attracted considerable interest for their anticancer properties (Barbosa et al. 2017; Garbo et al. 2023; Pyka et al. 2024; Yakubov et al. 2021; L. He et al. 2024). In parallel, ILs have emerged as promising scaffolds due to their tunable physicochemical properties and growing applications in biomedicine (Beaven et al. 2024; Novello et al. 2024; Alberto et al. 2011). Accordingly, this study evaluated the cytotoxic potential of ILsSe in human PBMCs, their interaction with Hg^2+^, their pharmacokinetic properties, and their antiproliferative activity in U87-MG glioblastoma cells.

The preparation of compound **2** was performed in a manner analogous to the one previously reported for compounds **1** and 3 (Alberto et al. 2012). The reaction sequence began with the bromination of diselenide **4** using HBr in acetic acid. In this step, bromide **5** was obtained in a quantitative yield. Lastly, treatment of **5** with 1-butylimidazole in THF delivered the target ILsSe **2** in 80% yield.

PBMCs play an important role regulating immune function and are widely used to investigate mechanisms of action of agents with immunological effects (Wildner et al. 2023; da Silva et al. 2024). Of pharmacological significance, Hg^2+^ is immunotoxic to mammals, and lymphocytes are targets of its cytotoxicity (Maqbool et al. 2017; Schott et al. 2025). In this work, we demonstrate that the ILsSe were generally well tolerated by PBMCs across most tested conditions, without significant effects on cell viability or intracellular dehydrogenase activity at low concentrations (1–10 μM). The absence of cytotoxicity in the Trypan Blue assay indicates preservation of membrane integrity, while the lack of major changes in MTT reduction suggests that cellular metabolic activity was largely maintained (Figures 2 and 3). However, a reduction in viability was observed for compound C3 at 10 μM after 72 h (Figure 2E), indicating a time- and concentration-dependent cytotoxic effect. In addition, compound C2 transiently decreased dehydrogenase activity at 10 μM after 24 h (Figure 3D), suggesting an early metabolic disturbance that was not sustained over prolonged exposure periods.

These findings are consistent with previous reports showing that certain Se-based compounds exhibit low toxicity toward normal human cells at comparable concentrations (Wildner et al. 2023; Golin et al. 2025). Taken together, these findings indicate that ILsSe are generally well tolerated by human PBMCs, but their biological effects appear to be highly dependent on both exposure time and concentration. This behavior is consistent with the well-known narrow therapeutic window of organoselenium compounds, in which small variations in dose or time exposition can shift the balance between beneficial and deleterious effects (Nogueira and Rocha 2011; Radomska et al. 2021). Therefore, although the results support the potential of ILsSe as biologically active molecules, careful consideration of experimental conditions is essential for their safe and effective application.

After assessing the toxicity of the compounds in human PBMCs, their potential interaction with Hg^2+^ was also explored, given that experimental studies have demonstrated that Se can bind to mercury, forming Se–Hg complexes and modulating the availability of Se for antioxidant enzymes such as glutathione peroxidase (Scheme 1B) (Barbosa et al. 2024, 2017; Tinggi and Perkins 2022). In cultured cells, simultaneous exposure to sodium selenite and HgCl_2_ has been shown to influence mercury-induced apoptosis, likely through direct Se–Hg interactions (Wang et al. 2001). Additionally, previous studies have highlighted the high affinity of Se for Hg exceeding that of sulfur, which may favor the formation of stable Se–Hg species (Oliveira et al. 2017; Raymond and Ralston 2020; Tinggi and Perkins 2022).

In the present study, our data indicate that the tested ILsSe do not exert a protective effect against Hg^2+^-induced cytotoxicity in PBMCs. Instead, the observed responses suggest that these compounds may interact with Hg^2+^, potentially leading to the formation of Se–Hg species under the experimental conditions. This interpretation is supported by the thiol oxidase assay (Figure 6), in which both DPDS and compound C3 accelerated the oxidation of DTT, which was completely blocked by Hg^2+^. These results indicated that diselenides can be reduced to their selenol intermediates and preferentially interact with mercury (Scheme 1B).

Accordingly, the formation of seleno–mercury complexes is likely to occur because diselenides can be reduced to their corresponding selenolate/selenol forms by enzymes such as thioredoxin reductase (TrxR) and by endogenous thiol-containing molecules, such as glutathione (GSH) (Nogueira et al. 2021; De Freitas and Rocha 2011). The selenolate species is highly nucleophilic and can react with electrophilic mercury compounds, leading to the formation of a stable adduct containing a Se–Hg bond (RSeSeR → RSe^−^; RSe^−^ + HgCl_2_ → RSeHgCl + Cl^−^; RSeHgCl + RSe^−^ → RSeHgSeR) (Gould and Amendola 1955; Konu and Chivers 2010; Barbosa et al. 2017). This seleno–mercury complex may act as a detoxifying agent, reducing mercury levels in the organism and facilitating its excretion. On the other hand, it is also possible that the formed complex acts as a mercury-delivery species via ligand exchange reactions, such as Rabenstein's reaction (RSeHgCl + R′S(e)H → R′S(e) – HgCl + RSeH) (Nogara et al. 2019; Madabeni et al. 2020; Barbosa et al. 2017). In this case, the complex could react with biologically important thiol(selenol)-containing proteins, leading to their inhibition and contributing to the toxic effects. Taken together, these findings support the hypothesis that ILsSe can interact with Hg^2+^ through the formation of Se–Hg species, although the biological consequences of this interaction appear to be context-dependent and require further investigation.

After assessing toxicity and interactions with mercury, we investigated the antiproliferative effects of these ILsSe in U87-MG human glioblastoma cells (U87-MG) (Figure 7). Notably, C3 at 50 μM significantly reduced glioblastoma U87-MG cell viability, and its efficacy persisted at concentrations as low as 10 μM, indicating concentration- and time-dependent antiproliferative effects (Figure 8). Indeed, IC_50_ values progressively decreased from 19.98 μM at 24 h to 12.29 μM at 48 h and 11.27 μM at 72 h (Figure 8). Consistently, 10 μM reduced glioblastoma cell viability across all evaluated time points, whereas no comparable effect was observed in PBMCs (Figures 2 and 3), supporting a degree of selectivity toward tumor cells. The superior toxicity of C3 may be related to the size of the alkyl portion bound to the imidazole ring, which could help stabilize interactions between C3 and proteins with hydrophobic pockets, although further studies are needed to confirm this mechanism. These findings are in line with previous studies showing that Se compounds exert anticancer effects in glioma models (Ferreira et al. 2019; Radomska et al. 2021; Berthier et al. 2017; Rieder et al. 2026). For instance, Se compounds have been reported to inhibit proliferation and induce apoptosis in glioblastoma cell lines, including U87-MG, at concentrations in the low micromolar range (Ferreira et al. 2019; Rooprai et al. 2007; Wang et al. 2016; Radomska et al. 2021). Various Se derivatives have been found to trigger apoptotic pathways and act in endoplasmatic reticulum stress (ER-stress) signaling, mechanisms implicated in tumor growth suppression (Markouli et al. 2020; Lee et al. 2015; Jehan et al. 2022; Rooprai et al. 2007; Xiang et al. 2009). Moreover, broader reviews emphasize Se's promise as a micronutrient therapeutic in glioblastoma treatment, highlighting its roles in redox balance regulation and tumor microenvironment modulation (Varlamova 2018; Radomska et al. 2021; Yakubov et al. 2021).

Consistently with these observations, our mechanistic analyses provide further insight into the biological effects of C3 in glioblastoma cells. Treatment with C3 reduced total thiol levels (Figure 10A), increased GPx activity (Figure 10B), and decreased intracellular ROS levels (Figure 11), indicating modulation of cellular redox homeostasis. Given the dependence of glioblastoma cells on adaptive antioxidant systems, these alterations suggest disruption of redox homeostasis and induction of redox vulnerability, potentially compromising cell survival (Ranbhise et al. 2025; Olufunmilayo et al. 2023; Liu and Liu 2025).

In addition, C3 altered cell cycle progression by affecting the G2/M phase and significantly increased late apoptosis/necrosis, without affecting early apoptotic events. This pattern suggests that C3 may preferentially promote cell death at later stages, possibly as a consequence of sustained metabolic and redox disturbances, as previously reported for redox-active anticancer agents that induce G2/M arrest and apoptosis (Nie et al. 2023; Sanmartín et al. 2012). Taken together, these findings indicate that the antiproliferative effects of C3 are associated with redox modulation, disruption of cell cycle progression, and induction of late-stage cell death in U87-MG cells.

Furthermore, for comparison purposes, we performed ADMET analyses on DPDS and derivatives. DPDS was included for comparison purposes because it is an organoselenium compound extensively studied in the literature (da Costa et al. 2023; Satoh et al. 2004; de Freitas et al. 2009). Compounds C1, C2, and C3 showed results similar to DPDS in terms of solubility, intestinal absorption, skin permeability, and interaction with different cytochrome P450 isoforms. Nevertheless, C1, C2, and C3 exhibited lower maximum tolerated concentration values compared to DPDS, which displayed lower LOAEL and LD_50_ values when compared to C1, C2, and C3. Additionally, the properties of the imidazole group were evaluated to determine whether it could confer key features to the new compounds. Overall, the imidazole group alone demonstrated absorption and toxicity profiles comparable to those of the tested compounds. On the other hand, imidazole did not interact with cytochrome P450 isoforms and showed a reduced ability to cross the BBB and penetrate the CNS.

Notably, C1, C2, and C3 were predicted to exhibit high BBB permeability and CNS penetration, which may represent a favorable feature for glioblastoma therapy, as it suggests the potential to reach tumor tissue in vivo (Van Tellingen et al. 2015; Arvanitis et al. 2020). This contrasts with the limited BBB permeability predicted for imidazole alone, highlighting the contribution of the overall molecular structure to this property.

In addition, the compounds were predicted to present a low MTD, which may indicate limited systemic tolerability and suggests the need for careful dose optimization in future studies. This feature could reflect a narrower therapeutic window, a common characteristic of biologically active compounds with cellular effects (Workman et al. 2010; Radomska et al. 2021). Importantly, considering that C3 demonstrated antiproliferative activity at low micromolar concentrations, effective doses may still fall within a tolerable range. Therefore, while the predicted low MTD highlights a potential limitation, it does not preclude the therapeutic potential of these compounds and warrants further in vivo evaluation.

Regarding safety-related parameters, the compounds were not predicted to inhibit hERG I, a key marker of cardiotoxic risk (Sanguinetti and Tristani-Firouzi 2006). However, a positive prediction for hERG II inhibition was observed, suggesting a potential interaction with cardiac ion channels. Therefore, this result should be interpreted with caution, as in silico predictions alone are not sufficient to establish cardiotoxic risk, although it warrants further experimental investigation (Ferri et al. 2013; Raies and Bajic 2016). Finally, it is important to note that all ADMET data presented here are based on in silico predictions (Raies and Bajic 2016). Experimental validation of key physicochemical and pharmacokinetic parameters, such as lipophilicity and solubility, will be required to confirm these findings.

In conclusion, ILsSe were generally well tolerated by human PBMCs at low micromolar concentrations, although their effects depended on exposure time and dose. The data suggest that ILsSe can interact with Hg^2+^, likely through the formation of Se–Hg species. Among the tested compounds, C3 showed antiproliferative effects in U87-MG glioblastoma cells, associated with alterations in redox balance, cell cycle progression, and induction of late-stage cell death. In silico analyses suggest that compound C3 may be capable of crossing the BBB and reaching the brain, although these predictions require experimental validation. Overall, the findings indicate that ILsSe are biologically active molecules whose effects are strongly influenced by experimental conditions, warranting further investigation to better define their mechanisms of action and potential applications. In this context, the present study highlights the importance of investigating Se-containing compounds and their biological effects.

## Figures and Tables

**TABLE 1 ∣:** IC_50_ values of selenium-containing ionic compounds (C1–C3) and HgCl_2_ in PBMCs. IC_50_ values (μM) were determined after 24, 48, and 72 h of exposure using the Trypan Blue exclusion assay.

Compound	IC50 value (μM)		
	24 h	48 h	72 h
**C1**	> 200	> 200	> 200
**C2**	197.30	138.5	118.5
**C3**	29.64	20.21	16.33
**HgCl_2_**	112.70	76.04	63.72

**TABLE 2 ∣:** ADMET analysis of compounds C1, C2, and C3. Abbreviations: VDss: steady-state volume of distribution. BBB: blood–brain barrier permeability. CNS: central nervous system permeability. CYP2D6/CYP3A4: cytochrome P450 substrate/inhibitor. Renal OCT2: renal organic cation transporter 2. hERG: potassium channel. AMES toxicity: predicted mutagenic potential.

ADMET prediction parameters	C1	C2	C3	DPDS	Imidazole
**Water solubility (log mol/L)**	−2.891 (medium)	−2.885 (medium)	−2.892 (medium)	−2.773 (medium)	−1.645 (high)
**Intestinal absorption (%)**	86.206 (high)	86.449 (high)	79.899 (high)	82.44 (high)	100 (high)
**Skin permeability (log Kp)**	−2.735 (low)	−2.735 (low)	−2.735 (low)	−1.958 (low)	−2.743 (low)
**P-glycoprotein substrate**	Yes	Yes	Yes	No	Yes
**P-glycoprotein I inhibitor**	Yes	Yes	Yes	No	No
**P-glycoprotein II inhibitor**	Yes	Yes	Yes	No	No
**VDss (log L/kg)**	0.043 (medium)	0.106 (medium)	0.031 (medium)	0.357 (medium)	−0.134 (medium)
**Fraction unbound (Fu)**	0.261	0.204	0.228	0.13	0.674
**BBB permeability (log BB)**	0.732 (high)	0.678 (high)	1.066 (high)	0.389 (high)	0.095 (medium)
**CNS permeability (log PS)**	−1.407 (high)	−1.439 (high)	−1.342 (high)	−1.314 (high)	−2.747 (medium)
**CYP2D6 substrate**	No	No	No	No	No
**CYP3A4 substrate**	Yes	Yes	Yes	Yes	No
**CYP1A2 inhibitor**	Yes	Yes	Yes	Yes	No
**CYP2C19 inhibitor**	Yes	Yes	Yes	No	No
**CYP2C9 inhibitor**	No	No	Yes	No	No
**CYP2D6 inhibitor**	Yes	Yes	Yes	Yes	No
**CYP3A4 inhibitor**	Yes	Yes	No	No	No
**Total clearance (log ml/min/kg)**	1.414	1.969	2.414	1.64	0.933
**Renal OCT2 substrate**	Yes	Yes	Yes	Yes	No
**AMES toxicity**	No	No	No	No	No
**Max tolerated dose (log mg/kg/day)**	0.385 (low)	0.396 (low)	0.372 (low)	0.689 (high)	−0.517 (low)
**hERG I inhibitor**	No	No	No	No	No
**hERG II inhibitor**	Yes	Yes	Yes	No	No
**Oral rat acute toxicity (LD50, mol/kg)**	2.479	2.526	2.47	1.721	3.192
**Oral rat chronic toxicity (LOAEL, log mg/kg_bw/day)**	1.926	2.002	1.745	0.645	1.095
**Hepatotoxicity**	No	No	No	No	No

## Data Availability

The data that support the findings of this study are available on request from the corresponding author. The data are not publicly available due to privacy or ethical restrictions.
